# The efficacy and safety of direct-acting antiviral drugs in the management of hepatitis C virus-related arthritis

**DOI:** 10.1186/s43166-020-00021-6

**Published:** 2020-09-29

**Authors:** Samah M. Alian, Mohamed Othman Wahba, Ahmed Fathy Gomaa, Sahar S. Khalil

**Affiliations:** 1 Rheumatology and Rehabilitation Department, Faculty of Medicine, Zagazig University, Zagazig, Egypt; 2 Internal Medicine Department, Faculty of Medicine, Zagazig University, Zagazig, Egypt; 3 Physical Medicine and Rehabilitation Department, Dubai hospital, Dubai, United Arab Emirates

**Keywords:** Hepatitis C virus, HCV-related arthritis, Management, Direct-acting antiviral drugs, Sofosbuvir, Daclatasvir

## Abstract

**Background:**

Hepatitis C virus (HCV) infection is a worldwide disease. HCV-related arthritis is one of the extrahepatic manifestations of the disease. The treatment of chronic HCV has been revolutionized with the introduction of oral direct-acting antiviral (DAA) drugs. We aim to determine the outcomes of treatment by the combination of sofosbuvir-daclatasvir with or without ribavirin in patients with HCV-related arthritis.

**Results:**

Post-therapy, all group I patients had sustained viral response. Significant improvement of the outcome parameters was found 12 weeks post-treatment in group I compared to baseline and group II. Complete and partial remission of articular symptoms in group I patients was observed in 80% and 5%, respectively, while 85% of patients in group II showed no remission. Few mild side effects were encountered with therapy.

**Conclusion:**

The combination of sofosbuvir-daclatasvir with or without ribavirin is an effective and safe therapy for eradication of HCV infection and amelioration of HCV-related arthritis.

## Background

Hepatitis C virus (HCV) infection is a worldwide disease affecting approximately 200 million people and considered a major cause of morbidity and mortality [1].

In Egypt, the extensive parenteral anti-schistosomal therapy treatment campaigns in the past era caused high rates of HCV transmission [2]. Several Egyptian demographic health surveys were conducted; they estimated the seroprevalence of HCV in Egyptian population, which was > 40% among adults in 1996 [3], reached 14.7% in 2008 [4], then dropped further to 10% in 2015 [5]. Accordingly, Egypt has the highest prevalence of HCV infection in the world [6, 7].

Hepatitis C virus affects the liver primarily; however, chronic HCV infection can cause several extrahepatic manifestations that can affect many systems, including skin, musculoskeletal, renal, cardiovascular, and nervous systems [8]. Extrahepatic manifestations develop in up to 74% of patients with chronic HCV infection during the disease course [9].

Musculoskeletal and rheumatologic extrahepatic manifestations associated with HCV infections are numerous and diverse including fatigue, myalgia, arthritis or arthralgia, mixed cryoglobulinemia, small and medium-sized vessel vasculitis, sicca syndrome, and fibromyalgia [10]. The actual prevalence of HCV-associated arthritis varies with a different population, but generally, in literature, it ranges from 4 to 12% of HCV-infected patients [10, 11].

Musculoskeletal ultrasound (MSUS) is a valuable tool for identifying any change in the joint even in the presence of active arthritis. It is a noninvasive, cheap, and sensitive method that can efficiently evaluate both articular and periarticular tissues [12].

The previous standard therapy of HCV was the combination of pegylated interferon and ribavirin, but this was associated with either worsening of preexisting autoimmune disorders or even developing a new one besides the poor virologic response in some cases [13–15].

The treatment of chronic HCV has been revolutionized with the introduction of the oral direct-acting antiviral (DAA) drugs, which specifically target virus-specific proteins. Sofosbuvir (SOF) and daclatasvir (DCV) are new generation DAA, which have proved high efficacy in treating HCV genotype 4 (the commonest genotype in Egypt), especially when used in combination. The

combination of SOF–DCV with or without ribavirin (RBV) has been entirely used for all patients in a national program adopted and completely funded by the Egyptian National Committee for Control of Viral Hepatitis (NCCVH) since 2016 [16, 17].

The aim of this study was to investigate the safety and efficacy of oral interferon-free, direct-acting antiviral therapy (SOF and DCV) with or without ribavirin (RBV) in patients with HCV-related arthritis compared to non-treated waiting list patients.

## Methods

### Study design and population

This study is a single-center, observational, prospective study, which enrolled forty consecutive Egyptian patients with concurrent HCV viremia and HCV-related arthritis, who were already assigned to receive the combination of SOF-DCV with or without RBV regimens as well as those who were in the waiting list of the same therapy during the period from June 2017 to December 2017. The study was carried out in both Rheumatology Department and Internal Medicine Department, Faculty of Medicine, …University, Egypt.

A total of 40 HCV patients with HCV-related arthritis were included in this study as follows: group I included 20 consecutive patients who were already assigned to receive the combination of sofosbuvir-daclatasvir (SOF-DCV) with or without ribavirin regimens, and group II included 20 consecutive patients who were included in the waiting list for the same therapy.

Informed consents were obtained from all the participants before entering the study. The study was approved by the ethics committee of the university and was conducted in accordance with the declaration of Helsinki.

Patients were included in this study if they were above 18 years and had concurrent HCV viremia and HCV-related arthritis confirmed clinically, laboratory, and radiological (MSUS). According to NCCVH guidelines, patients were excluded from DAA therapy if they had one of the following: Child’s C liver cirrhosis, positive hepatitis B surface antigen, positive human immunodeficiency virus test, platelet count < 50,000/mm3, hepatocellular carcinoma, malignancy within past 2 years, pregnancy, or ineffective contraception and uncontrolled systemic diseases, which are considered contraindication for anti-viral therapy, e.g., uncontrolled diabetes mellitus, advanced heart, and kidney diseases. Moreover, patients were excluded from the study if they had one of the following: concurrent autoimmune rheumatic diseases or arthritis associated with mixed cryoglobulinemia and lastly, the presence of radiologic bone erosions, joint deformities, and the positive anti-cyclic citrullinated peptide (anti-CCP) antibody test, as their presence favor the diagnosis of RA [18–21].

### Assessments

#### Clinical and laboratory assessments

All patients at both groups were subjected to detailed history taking, clinical musculoskeletal examination, laboratory investigations, and musculoskeletal ultrasonography prior to initiation of therapy and 12 weeks post-therapy. The clinical assessment included the patient’s global pain assessment on a numerical rate scale (NRS) for pain, duration of morning stiffness (MS) in minutes, swollen joint count (SJC), and tender joint count (TJC) [22]. The laboratory investigations included erythrocyte sedimentation rate at first hour (ESR), complete blood count (CBC), liver and kidney function tests, alpha-fetoprotein, prothrombin time (PT), the titers of rheumatoid factor (RF), and anti-cyclic citrullinated peptide (anti-CCP) antibodies by conventional laboratory methods. Viral load was quantitatively measured by real-time polymerase chain reaction (PCR) using “Cobas Ampliprep/Taqman HCV monitor version 2.0, with a detection limit of 15 IU/ml; Roche Diagnostic Systems, Pleasanton, California, USA”, and it was repeated 12 weeks after treatment to detect sustained virologic response (SVR12). All patients were subjected to full abdominal ultrasound scanning and liver examination. The severity of liver disease was determined by calculation of Child-Pugh score [23].

### Musculoskeletal US (MSUS)

Detailed MSUS examinations of the joints were done by a well-experienced rheumatologist with more than 5 years of experience in the MSUS field; he was blind to the clinical and laboratory data of the patients. B-Mode real-time ultrasound and Doppler ultrasound were performed using, “Hitachi-Aloka F37, Japan interfaced with a 10–18 MHz linear array transducer.” The examined joints were the clinically symptomatic joints. These joints were assessed according to the European League Against Rheumatism (EULAR) guidelines for musculoskeletal US examination, and the findings were documented following OMERACT definitions of MSUS pathologies [24, 25]. The abnormalities were scored semi-quantitatively using a (0–3) scale, based on the method first proposed by Szudlarek et al. [26], but taking into consideration the modification done by Scheel et al. [27], in respect of grey-scale synovitis, whereby effusion and synovial thickening were grouped to give a single combined score as the following:

1. Grey-scale ultrasound (GSUS): Synovitis was scored semi-quantitatively as follows: “grade 0 (absence), grade 1 (small hypoechoic/anechoic line beneath joint capsule), grade 2 (joint capsule elevated parallel to the joint area), and grade 3 (strong distension of joint capsule)”.
2. Power Doppler ultrasound (PDUS): PDUS activity findings were scored semi-quantitatively as follows: “grade 0 = no intra-articular colour signal; grade 1 = up to three single colour signals or two single-colour signals and one confluent colour signal representing only low flow; grade 2 = < 50% of the intra-articular area filled with colour signals representing clear flow; and grade 3 = 50% of the intra-articular area filled with colour signals”.

### Therapy

#### Protocol

All patients at group I had received the combination of sofosbuvir 400 mg once daily and daclatasvir 60 mg once daily. We added to the previous combination ribavirin for patients with the following parameters: total bilirubin ˃ 1.2 mg/dl, serum albumin < 3.5 gm/dl, INR ˃ 1.2, and platelet count < 150,000. The dose of ribavirin was 1200 mg daily if weight > 75 kg or 1000 mg daily if weight ≤ 75 kg. The therapy course duration was 12 weeks. This treatment regimen is complying with the protocol of the Egyptian National Committee for Control of Viral Hepatitis (NCCVH) [28]. All patients were asked to stop all arthritis medications before entering the study and were allowed to use only oral paracetamol up to 2 g daily if needed.

#### Therapy follow-up

All patients at both groups were followed up by assessing liver function tests, bilirubin level, CBC, and blood creatinine level at weeks 4, 8, and 12, respectively, to detect any complication of therapy. Quantitative PCR was done after treatment completion to detect the end of treatment response (ETR) and after 12 weeks to evaluate sustained virologic response (SVR12). Any potential drug-related adverse events were supervised during the treatment period.

### Outcome measures

Outcomes of therapy on joints were evaluated by the following: 28-tender joints count (TJC), 28-swollen joints count (SJC), patient’s global assessment of pain on a numerical rate scale (NRS) for pain, morning stiffness (MS) duration in minutes, ESR at the first hour, GSUS synovitis, and PDUS scores. Patients were assessed prior to treatment initiation as well as 12 weeks after it.

Articular remission was assessed 12 weeks after the end of treatment using all the previously mentioned outcome parameters except the RF titer. Remission was defined as the following: complete remission was defined as improvement in all baseline outcome parameters and absence of clinical relapse, while partial remission was defined as improvement in at least one half of baseline outcome parameters. No remission was defined in patients who did not fulfill the criteria for any of the previous two conditions.

### Statistical methods

All data were collected and analyzed using the Statistical Package for Social Sciences (SPSS) version 25. Quantitative data were expressed as the mean ± SD, and qualitative data were expressed as number and percentage. Percentage of categorical variables was compared using chi-square test when appropriate. Comparison of quantitative data between groups both parametric and non-parametric distribution was done using “Independent t-test or Mann– Whitney test”, respectively. The continuous variables across time either parametric or non-parametric were compared using “paired t-test or Wilcoxon Signed-Rank test”, respectively. All tests were two-sided differences and were considered statistically significant when *P* values were < 0.05.

## Results

### Baseline characteristics of the study population

This study was conducted on forty HCV-related arthritis patients, 20 patients in each group. There was no significant difference in all baseline demographic, clinical, pre-study ultrasonographic, and medications history between the two groups (*p* > 0.05) (Table 1).

**Table 1:** Baseline characteristics (demographic, clinical, laboratory, ultrasonographic, and pre-study medications) of both groups

Baseline characteristics		Group I (*n* = 20)	Group 1I (*n* = 20)	*p*
Age (mean ± SD)		43.5 ± 7.4	43.8 ± 7.2	0.19
Male: female (*n*, %)		10(50):10(50)	9 (45):11 (55)	0.75
Disease duration (mean ± SD)		3.1 ± 1.6	3.0 ± 1.4	0.95
RF titer (mean ± SD)		101.2 ± 76.2	99.6 ± 73.1	0.65
Viral load (X106) (mean ± SD)		7.04 ± 8.02	6.76 ± 7.47	0.16
ESR 1st hour		52.2 ± 19.0	52.0 ± 16.7	0.78
NRS for pain (mean ± SD)		6.3 ± 2.9	6.2 ± 3.1	0.64
TJC (mean ± SD)		9.8 ± 9.4	10.2 ± 10.6	0.07
SJC (mean ± SD)		7.3 ± 7.9	7.3 ± 8.2	0.98
Morning stiffness (MS) (mean ± SD)		68.9 ± 22.7	68.5 ± 26.5	0.19
GSUS synovitis score (mean ± SD)		1.8 ± 0.8	1.9 ± 0.8	0.41
PDUS score (mean ± SD)		0.9 ± 0.8	0.9 ± 0.8	1.00
Pre-study medications, *n* (%)	Analgesics^#^	15 (75)	14 (70)	0.72
	Steroid^+^	12 (60)	13 (65)	0.74
	DMARDs^*^	9 (45)	11 (55)	0.52

#Analgesics used were paracetamol and tramadol

+Steroid dosage ranged from 5 to 20 mg/day

*DMARDs used were sulfasalazine and cyclosporine

*RF* rheumatoid factor, *ESR* erythrocyte sedimentation rate, *NRS* numerical rate scale, *TJC* tender joints count, *SJC* swollen joints count, *MS* duration of morning stiffness, *GSUS* grey scale ultrasound, *PDUS* Power Doppler ultrasound, *DMARDs* disease modifying anti-rheumatic drugs

### Pattern and distribution of articular involvement

Most of the patients of both groups had symmetrical polyarthritis (65% in group I and 60% in group II). The most frequent joints involved in both groups were the ankle, MCP, PIP, and wrist joints. No significant differences were found between both groups in the pattern and distribution of the articular manifestations (*P* > 0.05) (Table 2).

**Table 2:** Joints involvement and pattern in the study groups

Outcome variables (*n*, %)		Group I (*n* = 20)	Group 1I (*n* = 20)	*P*
Arthritis pattern	Symmetrical polyarthritis	13 (65)	12 (60)	0.74
	Oligo- or mono-arthritis	7 (35)	8 (40)	
Ankle involvement		16 (80)	16 (80)	1.00
Knee involvement		7 (35)	6 (30)	0.74
Hip involvement		8 (40)	7 (35)	0.75
Wrist involvement		12 (60)	14 (70)	0.51
Elbow involvement		8 (40)	8 (40)	1.00
Shoulder involvement		10 (50)	8 (40)	0.52
MCP involvement		14 (70)	14 (70)	1.00
PIP involvement		13 (65)	14 (70)	0.74

*MCP* metacarpophalangeal joint, *PIP* proximal interphalangeal joint, *MTP* metatarsophalangeal

Twelve weeks after the end of treatment, there was a significant improvement in all measured outcomes (clinical, laboratory, and ultrasonographic) in group I compared to baseline and group II (*p* < 0.05). There was no significant improvement in the outcome measures in group II compared to baseline at the follow-up (*p* > 0.05) (Table 3).

**Table 3:** Outcome measures before and after treatment in the study groups

Outcome scales	Groups	Baseline (0 weeks)	12 weeks after treatment end	*p* value^2^
NRS for pain (mean ± SD)	I II *p* value^1^	6.2 ± 3.1 6.3 ± 2.9 0.64	1.7 ± 3.5 5.7 ± 3.1 0.000*	0.000* 0.10
MS, min (mean ± SD)	I II *p* value^1^	68.5 ± 26.5 68.9 ± 22.7 0.19	13.2 ± 31.7 63.5 ± 27.8 0.000*	0.000* 0.14
TJC (mean ± SD)	I II *p* value^1^	10.2 ± 10.6 9.8 ± 9.4 0.07	4.2 ± 9.7 9.4 ± 9.7 0.000*	0.000* 0.10
SJC (mean ± SD)	I II *p* value^1^	7.3 ± 8.2 7.3 ± 7.9 0.98	3.2 ± 8.0 6.9 ± 8.1 0.000*	0.000* 0.06
ESR 1st hour (mean ± SD	I II *p* value^1^	52.0 ± 16.7 52.2 ± 19.0 0.78	19.2 ± 22.3 48.2 ± 23.5 0.000*	0.000* 0.06
RF titer (mean ± SD)	I II *p* value^1^	99.6 ± 73.1 101.2 ± 76.2 0.65	45.1 ± 62.4 96.0 ± 80.1 0.000*	0.000* 0.07
GSUS synovitis score (mean ± SD)	I II *p* value^1^	1.9 ± 0.8 1.8 ± 0.8 0.41	0.7 ± 1.1 1.5 ± 0.8 0.02*	0.000* 0.11
PDUS score (mean ± SD)	I II *p* value^1^	0.9 ± 0.8 0.9 ± 0.8 1.00	0.3 ± 0.73 0.95 ± 0.82 0.01*	0.006* 0.86

*P* value^1^ Significance before and after treatment between both groups *P* value^2^ Significance before and after treatment in the same group

*RF* rheumatoid factor, *ESR* erythrocyte sedimentation rate, *NRS* numerical rate scale, *TJC* tender joints count, *SJC* swollen joints count, *MS* duration of morning stiffness, *GSUS* grey scale ultrasound, *PDUS* Power Doppler ultrasound

* means statistically signifiicant *P* value

#### Virologic and articular response to therapy

Regarding the virologic response, all group I patients had undetectable HCV RNA (100%) after completion of therapy (ETR). Moreover, this response was sustained for 12 weeks after (SVR12), with no relapse recorded. However, group II patients had no significant change in HCV RNA level at any follow-up point (not presented in table).

**Fig. 1:**
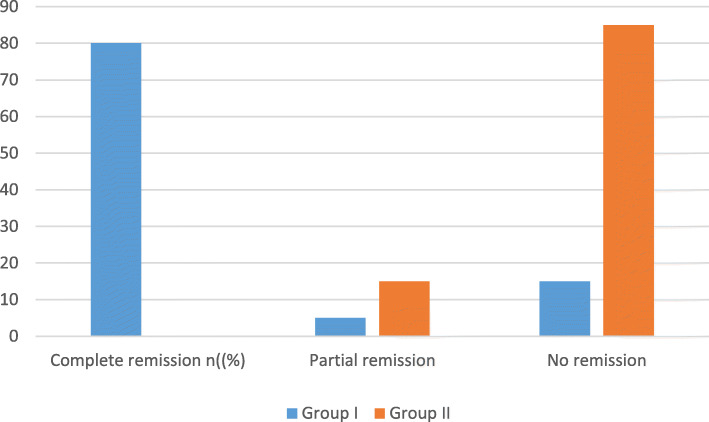
Remission of articular symptoms in both groups (12 weeks post-therapy) (*P* < 0.05)

Concerning articular remissions, in group I, complete and partial remissions of articular symptoms were observed in 80% and 5%, respectively, while 15% had no remission in their symptoms. In group II, partial remission was observed in 15%, while 85% had no remission. The difference between both groups was statistically significant (*p*˂ 0.05) (Fig. 1).

### Therapy-related adverse events

**Fig. 2:**
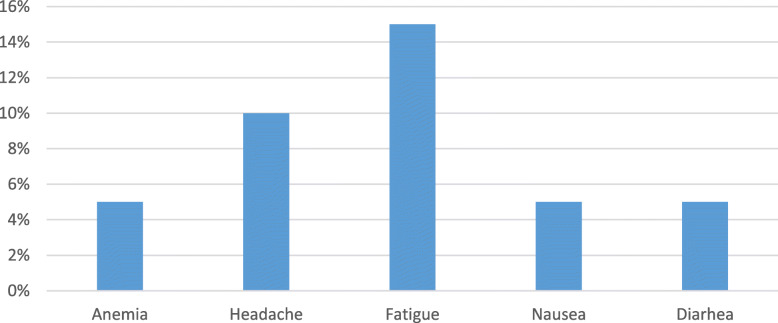
Therapy-related adverse effects among the treated group

No major side effects were encountered. Few mild side effects were encountered with therapy, were fatigue (15%), and headache (10%) being the most frequent (Fig. 2). All group I patients completed the treatment course, and all the patients completed their follow-up appointment.

## Discussion

Since the introduction of new direct-acting antiviral agents (DAAs) and its funding by the Egyptian government, the previous therapy with interferon has been completely stopped. There are few studies that have investigated the efficacy and safety of these drugs on HCV-related extra-hepatic manifestations [13, 29–31].

In our study, all group I patients had undetectable HCV RNA (100%) after completion of therapy (ETR), and this was sustained up to 12 weeks post-therapy (SVR12). However, group II patients had no significant change in HCV RNA level at 12 weeks follow-up period. The SVR12 range of 90–100% was noticed in several other previous studies [13, 29, 32].

At the follow-up, most of the patients in the treated group (group I) had entered into complete remission of the articular symptoms in comparison to the control group, in which no patient had entered into complete remission. On the other hand, three patients in the treatment group (15%) showed no remission in their articular symptoms that necessitated reuse of disease modifying anti-rheumatic drugs (DMARDs). We do not know whether this was a true failure of the antiviral therapy on their arthritis or they were actually having another form of arthritis, e.g., seronegative rheumatoid arthritis or bilharzial parasitic arthritis. Also, it is thought to be due to local inflammatory response to synovial tissue damage caused by viral invasion or indirectly by deposition of cryglobulin-induced immune complexes in synovial fluid [19]. We did not investigate the risk factors linked to this treatment non-response due to their small number, but we had some observations on them; all of those patients had long disease duration, high RF titer, moderate elevation of ESR and CRP levels, and history of using steroid with DMARDs before entering the study.

In this study, most of the patients had symmetrical polyarthritis (65% in group I and 60% in group II); the most frequent joints involved were the ankle, metacarpophalangeal (MCP), proximal interphalangeal (PIP), and wrist joints. No significant differences were found between both groups in the pattern and distribution of the articular manifestations.

In agreement with us, most of the researches which studied HCV-related arthritis reported that the most common pattern is the symmetric polyarthritis which resembles rheumatoid arthritis and the other less common pattern is oligoarthritis [33–36].

In addition, consistent with our results, Zukerman et al. [33] found in their study that the most common pattern of HCV-related is symmetric polyarthritis (68%). Moreover, they found the most commonly involved joints in polyarthritis patients were the MCP, PIP, wrists, and ankles, whereas, in oligoarthritis patients, the most commonly involved joints were the wrists, shoulders, ankles, and knees. According to the best of our knowledge, no study until now has used MSUS in HCV-related arthritis. In one study, they investigated US finding in HCV patients without clinical arthritis to detect any joint affection; they found the presence of joint changes in most asymptomatic HCV patients [12].

There is a paucity of studies that investigated the effects of sofosbuvir-daclatasvir with or without ribavirin therapy on HCV-related arthritis. Another Egyptian study was done on HCV patients with extra-hepatic manifestations compared to treatment by sofosbuvir-based therapy to a historic group who received interferon-based therapy. They found that all articular symptoms (NRS, MS, SJC, TJC) had significantly improved in sofosbuvir-treated group after treatment completion, while the SJC which decreased initially returned back to baseline values 24 weeks after treatment completion [29].

Another clinical study done by HCV-related mixed cryoglobulinemia found that the arthropathy frequency was 58%, and it had completely disappeared after completion of treatment with sofosbuvir/ribavirin therapy [13].

A retrospective cohort study included 121 American Veterans with HCV who received DAA therapy and were visiting one rheumatology clinic for joints pain (arthralgia/arthritis). There was moderate improvement in subjective pain scores and mild reduction in opioid prescriptions. However, this study had several limitations as it was retrospective and didn’t included objective outcome measures like tender and swollen joints count [37].

Concerning the therapy-related adverse effects in this study, the total side-effect frequency was 10%. These adverse effects were mostly mild and did not necessitate treatment cessation or modification of its course. The most common side effects were fatigue followed by headache.

In line with us, another study has used the same combination of therapy on HCV patients; it reported that only 19.7% of their patients had adverse events. All the adverse events were generally mild; the commonest were fatigue and anaemia in 9% and 5.67% of the patients, respectively [38].

The frequency of adverse events for second-generation DAA drugs in different studies ranged from 58 to 67%, higher than this study [13, 30, 31]. Of note, this may be attributed to different selection of inclusion criteria, like including patients with HCV-related mixed cryoglobulinemia. The first limitation of this study was the small number of patients included which is attributed to the selection of patients with only true arthritis, not arthralgia, and to the recent introduction of DAA therapy to Egypt and its sponsorship by the Egyptian government. Hence, we recommend further studies with larger numbers of HCV-related arthritis patients. The second limitation was the short follow-up duration period consequently, and we could not confirm whether remission would be sustained or there will be potential relapses with longer follow-up periods, so we recommend future studies to the group of patients who failed to get remission on antiviral treatment with full immunological investigations aiming to reach proper diagnosis and plan for their management.

## Conclusion

Sofosbuvir-daclatasvir with or without ribavirin combination therapy is an effective treatment for eradication of HCV infection and amelioration of HCV-related arthritis. It is considered effective and safe with minimal side effects.

## Data Availability

Not applicable

## Abbreviations

HCV: Hepatitis C virus
DAA: Direct-acting antiviral
MSUS: Musculoskeletal ultrasound
SOF: Sofosbuvir
DCV: Daclatasvir
DCV: Sofosbuvir-daclatasvir
RBV: Ribavirin
NCCVH: Egyptian National Committee for Control of Viral Hepatitis
anti-CCP: Anti-cyclic citrullinated peptide
NRS: numerical rate scale
MS: Morning stiffness
SJC: Swollen joint count
TJC: Tender joint count
ESR: Erythrocyte sedimentation rate
CBC: Complete blood count
PT: Prothrombin time
Rf: Rheumatoid factor (RF)
PCR: Polymerase chain reaction
SVR12: Sustained virologic response
EULAR: European League Against Rheumatism
OMERACT: Outcome measures in rheumatology clinical trials
GSUS: Grey-scale ultrasound
PDUS: Power Doppler ultrasound
ETR: End of treatment response
DMARDs: Disease modifying anti- rheumatic drugs (DMARDs)
MCP: Metacarpophalangeal
PIP: Proximal interphalangeal (PIP)
